# Malnutrition and visceral obesity predicted adverse short-term and long-term outcomes in patients undergoing proctectomy for rectal cancer

**DOI:** 10.1186/s12885-023-11083-y

**Published:** 2023-06-22

**Authors:** Chong-Jun Zhou, Yi Lin, Jie-Yu Liu, Zhong-Lin Wang, Xi-Yi Chen, Chen-Guo Zheng

**Affiliations:** 1 Department of Anorectal Surgery, The Second Affiliated Hospital, Yuying Children’s Hospital of Wenzhou Medical University, No.109 Xueyuan Road, Wenzhou, 325000 Zhejiang China; 2 Department of Endocrinology, The Wenzhou Third Clinical Institute, The Third Affiliated Hospital of Shanghai University, Wenzhou Medical University, Wenzhou People’s Hospital, No. 57 Canghou Street, Wenzhou, 325000 Zhejiang China; 3 Department of Cardiovascular and Thoracic Surgery, The Second Affiliated Hospital, Yuying Children’s Hospital of Wenzhou Medical University, No.109 Xueyuan Road, Wenzhou, 325000 Zhejiang China

**Keywords:** Visceral obesity, GLIM, Malnutrition, Rectal cancer, Adverse outcomes

## Abstract

**Background:**

To the best of our knowledge, no previous studies have explored the relationship between visceral obesity and malnutrition. Therefore, this study has aimed to investigate the association between them in patients with rectal cancer.

**Methods:**

Patients with rectal cancer who underwent proctectomy were included. Malnutrition was defined according to the Global Leadership Initiative on Malnutrition (GLIM). Visceral obesity was measured using computed tomography (CT). The patients were classified into four groups according to the presence of malnutrition or visceral obesity. Univariate and multivariate logistic regression analyses were performed to evaluate risk factors for postoperative complications. Univariate and multivariate cox regression analyses were performed to evaluate the risk factors for overall survival (OS) and cancer-specific survival (CSS). Kaplan-Meier survival curves and log-rank tests were performed for the four groups.

**Results:**

This study enrolled 624 patients. 204 (32.7%) patients were included in the well-nourished non-visceral obesity (WN) group, 264 (42.3%) patients were included in the well-nourished visceral obesity (WO) group, 114 (18.3%) patients were included in the malnourished non-visceral obesity (MN) group, and 42 (6.7%) patients were included in the malnourished visceral obesity (MO) group. In the multivariate logistic regression analysis, the Charlson comorbidity index (CCI), MN, and MO were associated with postoperative complications. In the multivariate cox regression analysis, age, American Society of Anesthesiologists (ASA) score, tumor differentiation, tumor node metastasis (TNM), and MO were associated with worsened OS and CSS.

**Conclusions:**

This study demonstrated that the combination of visceral obesity and malnutrition resulted in higher postoperative complication and mortality rates and was a good indicator of poor prognosis in patients with rectal cancer.

## Introduction

Malnutrition is a major health concern worldwide, as nutritional status is an important prognostic factor in patients with cancer [1]. Patients with cancer-related malnutrition experience increased postoperative complication and mortality rates [2]. Malnutrition has been responsible for 10–20% of deaths in patients with cancer rather than the tumor itself, because of the effect on the progression and therapeutic responses of cancer [3, 4]. Thus, the nutritional status of patients with cancer should be assessed, and nutritional interventions should be provided perioperatively, when necessary.

Pervasive obesogenic lifestyles have led to obesity epidemics worldwide [5]. Obesity is characterized by an increase in adiposity, which adversely affects health and is typically defined using body mass index (BMI) [6]. However, clinicians were unable to differentiate between visceral, subcutaneous, intermuscular, and intramuscular adiposity using BMI because it is a generalized measure and does not consider the composition of each body compartment. Therefore, individuals with obesity were highly heterogeneous [7, 8]. Visceral obesity, the accumulation of visceral adipose tissue (VAT), which is considered a more reliable indicator of obesity than BMI. [9, 10]. Visceral obesity had a negative impact on the outcomes of patients with cancer, including longer operative time, greater intraoperative blood loss, longer hospital stay, higher postoperative complications, and higher mortality rate [11, 12].

According to the World Health Organization (WHO), “a double burden of malnutrition” existed when undernutrition, obesity, and diet-related non-communicable diseases coexist, which is a real and growing global health challenge [13]. Some studies have explored the impact of malnutrition on obese patients’ outcomes, but BMI has remained the only surrogate measure for obesity. [14–16]. To the best of our knowledge, no previous studies have explored the relationship between visceral obesity and malnutrition. Therefore, this study has aimed to investigate the association between them in patients with rectal cancer.

## Material & Methods

### Patients

This study included patients with rectal cancer who underwent proctectomy between February 2013 and March 2019 at the Anorectal Surgery Department of the Second Affiliated Hospital of Wenzhou Medical University. Depending on the distance between the tumor and the rectum, the Dixon or Miles operation was performed. Inclusion criteria: (1) age ≥ 18 years; (2) American Society of Anesthesiologists (ASA) grade ≤ III; and (3) planning to undergo curative proctectomy. Exclusion criteria: (1) an available preoperative abdominal computed tomography (CT) beyond a month; (2) missing data; (3) undergoing palliative or emergency surgery; (4) receiving neoadjuvant treatments; or (5) patients with metastatic disease (stage IV). The data collection protocol for this study was approved by the Ethics Committee of the Second Affiliated Hospital of Wenzhou Medical University (LCKY2020–209) and complied with the Declaration of Helsinki. Informed consents were obtained from all participants.

### Data collection

Data were collected as follows: (1) general features, including age, gender, BMI, skeletal muscle index (SMI), visceral fat area (VFA), Charlson comorbidity index (CCI) [17], ASA grade, and previous abdominal surgery; (2) clinicopathological features, including tumor size, tumor location, tumor differentiation, tumor stage, node stage, and pathological tumor node metastasis (TNM) stage; and (3) postoperative short-term and long-term outcomes, including postoperative complications (according to the Clavien–Dindo classification grade [18]), postoperative hospital stay and mortality.

### Assessment of SMI and VFA

Preoperative abdominal CT images at the level of the third lumbar vertebra were obtained using specialized imaging software (INFINITT Healthcare Co., Ltd.) to calculate muscle mass, which was defined as the area shown in − 29 to + 150 Hounsfield unit (HU). VFA was defined as the area shown in − 150 to − 50 HU. The SMI was calculated as the area of muscle mass divided by the square of the height (m). Low SMI were determined by our previous study, < 40.8 cm^2^/m^2^ for males or < 34.9 cm^2^/m^2^ for females [19]. Visceral obesity was defined as VFA ≥ 100 cm^2^ in both males and females [20].

### Assessment of malnutrition

Malnutrition was diagnosed using the two-step model, Global Leadership Initiative in Malnutrition (GLIM) [21]. The first step was a Nutritional Risk Screening 2002 (NRS 2002) ≥ 3 to identify the individuals at risk of malnutrition. Second, malnutrition was defined if one of the three phenotypical criteria (non-volitional weight loss, low BMI and reduced muscle mass) was met as the patients in this study had already met the etiological criterion (disease burden) [21]. Non-volitional weight loss was defined as weight loss > 5% within 6 months or > 10% beyond 6 months. A low BMI score was defined when patients aged ≥ 70 years old had a BMI score of < 18.5 kg/m^2^, or when patients aged < 70 years old had a BMI score of < 20 kg/m^2^. Reduced muscle mass was defined as low SMI.

### Follow-up

Follow-up with patients via telephone was regularly conducted 1 month after surgery, every 3 months for 2 years, and every 6 months thereafter until death, or the end of the study in August 2022, or more than 8 years. Overall survival (OS) was calculated from the date of surgery until death.

### Statistical analysis

Regarding continuous variables, the mean and standard deviation (SD) or the median and interquartile range (IQR) were shown. Analysis of variance or Kruskal-Wallis H tests were used to compare continuous variables. Categorical variables were presented as numbers and proportions and Chi-squared or Fisher’s exact tests were used to compare them. Univariate and multivariate logistic regression analyses were performed to evaluate the relationship between the factors and postoperative complications. Univariate and multivariate cox regression analyses were performed to evaluate the risk factors for OS and cancer-specific survival (CSS). Kaplan-Meier survival curves and log-rank tests were performed for the four groups. Multivariate analyses were conducted on factors with P < 0.10 in the univariate analyses. Two-sided P < 0.05 was statistically significant. SPSS 26.0 and R software (version 4.2.1, https://cran.r-project.org) were used.

## Results

A total of 624 patients with rectal cancer were enrolled. Based on the diagnostic criteria, 306 (49.0%) and 156 (25.0%) patients were identified as having visceral obesity and malnutrition, respectively. The patients were classified into four groups according to their nutritional and visceral obesity status: 204 (32.7%) in the well-nourished non-visceral obesity (WN) group, 264 (42.3%) in the well-nourished visceral obesity (WO) group, 114 (18.3%) in the malnourished non-visceral obesity (MN) group, and 42 (6.7%) in the malnourished visceral obesity (MO) group.

Table 1 showed the clinical characteristics of the patients with rectal cancer in each group. Among the four groups, there were significant differences in age (P < 0.001), SMI (P < 0.001), VFA (P < 0.001), BMI (P < 0.001), CCI (P = 0.001), and ASA grade (P < 0.001). No significant differences in gender, previous abdominal surgery, tumor size, tumor location, tumor differentiation, tumor stage, node stage, TNM stage, and chemotherapy among the four groups.

**Table 1:** The patients’ clinical characteristics

Characteristics	Overall (n = 624)	WN (n = 204)	WO (n = 264)	MN (n = 114)	MO (n = 42)	*P*
Age, median (IQR), years	65 (58–74)	61 (53–67)	65 (58–73)	72 (63–77)	71 (62–80)	< 0.001^*^
Gender						0.084
Female	245 (39.3)	88 (43.1)	89 (33.7)	52 (45.6)	16 (38.1)	
Male	379 (60.7)	116 (56.9)	175 (66.7)	62 (54.4)	26 (61.9)	
SMI, mean (SD), cm^2^/m^2^	42.61 (8.53)	42.76 (7.67)	45.02 (8.31)	37.37 (7.37)	41.00 (10.27)	< 0.001^*^
VFA, median (IQR), cm^2^	98.9 (54.7–142.1)	64.8 (39.1–83.7)	143.9 (120.0–185.1)	38.6 (18.7–67.7)	135.0 (108.5–160.8)	< 0.001^*^
BMI						< 0.001^*^
< 18.5	72 (11.5)	0 (0)	0 (0)	65 (57.0)	7 (16.7)	
18.5–23.9	362 (58.0)	165 (80.9)	123 (46.6)	48 (42.1)	26 (61.9)	
≥ 24	190 (30.5)	39 (19.1)	141 (53.4)	1 (0.9)	9 (21.4)	
CCI						0.001^*^
0	427 (68.4)	154 (75.5)	158 (59.8)	87 (76.3)	28 (66.7)	
≥ 1	197 (31.6)	50 (24.5)	106 (40.2)	27 (23.7)	14 (33.3)	
ASA grade						< 0.001^*^
I	63 (10.1)	31 (15.2)	16 (6.1)	15 (13.1)	1 (2.4)	
II	460 (73.3)	155 (76.0)	204 (77.3)	75 (65.8)	26 (61.9)	
III	101 (16.2)	18 (8.8)	44 (16.6)	24 (21.1)	15 (35.7)	
Previous abdominal surgery						0.396
No	563 (90.2)	187 (91.7)	240 (90.9)	98 (86.0)	38 (90.5)	
Yes	61 (9.8)	17 (8.3)	24 (9.1)	16 (14.0)	4 (9.5)	
Tumor size, median (IQR), cm	4.0 (3.0–5.0)	4.0 (3.0–5.0)	4.0 (3.0–5.0)	4.0 (3.3–5.5)	4.5 (4.0-5.6)	0.064
Tumor location						0.544
Upper	487 (78.0)	166 (81.4)	202 (76.5)	86 (75.4)	33 (78.6)	
Lower	137 (22.0)	38 (18.6)	62 (23.5)	28 (24.6)	9 (21.4)	
Tumor differentiation						0.646
Well differentiated	543 (87.0)	177 (86.8)	230 (87.1)	97 (85.1)	39 (92.9)	
Poorly differentiated	81 (13.0)	27 (13.2)	34 (12.9)	17 (14.9)	3 (7.1)	
Tumor stage						0.739
Tis, T1	57 (9.2)	15 (7.4)	28 (10.6)	11 (9.6)	3 (7.1)	
T2	155 (24.8)	58 (28.4)	66 (25.0)	24 (21.1)	7 (16.7)	
T3	345 (55.3)	109 (53.4)	143 (54.2)	65 (57.0)	28 (66.7)	
T4	67 (10.7)	22 (10.8)	27 (10.2)	14 (12.3)	4 (9.5)	
Node stage						0.621
N0	366 (58.7)	113 (55.4)	160 (60.6)	66 (57.9)	27 (64.3)	
N1	155 (24.8)	57 (27.9)	62 (23.5)	25 (21.9)	11 (26.2)	
N2	103 (16.5)	34 (16.7)	42 (25.9)	23 (20.2)	4 (9.5)	
TNM stage						0.201
Tis, I	173 (27.7)	56 (27.5)	79 (29.9)	31 (27.2)	7 (16.7)	
II	190 (30.5)	55 (27.0)	80 (30.3)	35 (30.7)	20 (47.6)	
III	261 (40.8)	93 (45.5)	105 (39.8)	48 (42.1)	15 (35.7)	
Chemotherapy						0.707
No	339 (54.3)	105 (51.5)	144 (54.5)	66 (57.9)	24 (57.1)	
Yes	285 (45.7)	99 (48.5)	120 (45.5)	48 (42.1)	18 (42.8)	

Values in parentheses are percentages

^*^Statistically significant (*P* < 0.05)

IQR, interquartile range; SD, standard deviation; SMI, skeletal muscle index; VFA, Visceral fat area; BMI, body mass index; CCI, Charlson Comorbidity Index; ASA, American Society of Anesthesiologists; TNM, tumor node metastasis; WN, well-nourished non-visceral obesity; WO, well-nourished visceral obesity; MN, malnourished non-visceral obesity; MO, malnourished visceral obesity

Table 2 showed the details of postoperative complications and postoperative hospital stay. The overall postoperative complication rate was 23.2% (n = 145). The postoperative complication rates in the WN, WO, MN, and MO groups were 17.6% (n = 36), 21.2% (n = 56), 28.9% (n = 33), and 47.6% (n = 42), respectively, with a significant difference (P < 0.001). No significant difference in postoperative hospital stay among the four groups (P = 0.194) was noted.

**Table 2:** Postoperative outcomes

	Overall (n = 624)	WN (n = 204)	WO (n = 264)	MN (n = 114)	MO (n = 42)	*P*
^a^Total complications	145 (23.2)	36 (17.6)	56 (21.2)	33 (28.9)	20 (47.6)	< 0.001^*^
^b^Severe complications	23 (3.7)	6 (2.9)	9 (3.4)	5 (4.4)	3 (7.1)	0.583
Detail of complications						
Surgical complications	69 (11.1)	22 (10.8)	30 (11.4)	10 (8.8)	7 (16.7)	0.575
Intra-abdominal infection	13 (2.1)	5 (2.5)	4 (1.5)	2 (1.8)	2 (4.8)	0.554
Wound infection	17 (2.7)	5 (2.5)	8 (3.0)	2 (1.8)	2 (4.8)	0.750
Bleeding	5 (0.8)	2 (1.0)	3 (1.1)	0 (0.0)	0 (0.0)	0.634
Anastomotic leakage	10 (1.6)	2 (1.0)	6 (2.3)	1 (0.9)	1 (2.4)	0.616
Ileus	8 (1.3)	3 (1.5)	3 (1.1)	2 (1.8)	0 (0.0)	0.838
Gastrointestinal dysfunction	11 (1.8)	2 (1.0)	4 (1.5)	3 (2.6)	2 (4.8)	0.332
Ureteral fistula	2 (0.3)	1 (0.5)	1 (0.4)	0 (0.0)	0 (0.0)	0.870
Urinary retention	3 (0.5)	2 (1.0)	1 (0.4)	0 (0.0)	0 (0.0)	0.599
Medical complications	76 (12.2)	14 (6.9)	26 (9.8)	23 (20.2)	13 (31.0)	< 0.001^*^
Pulmonary complications	7 (1.1)	3 (1.5)	3 (1.1)	0 (0.0)	1 (2.4)	0.548
Cardiac complications	5 (0.8)	0 (0.0)	1 (0.4)	1 (0.9)	3 (7.1)	< 0.001^*^
Anemia	11 (1.8)	2 (1.0)	3 (1.1)	5 (4.4)	1 (2.4)	0.114
Persistent hypoalbuminemia	15 (2.4)	0 (0.0)	5 (1.9)	6 (5.3)	4 (9.5)	< 0.001^*^
Cerebral infarction	2 (0.3)	0 (0.0)	0 (0.0)	2 (1.8)	0 (0.0)	0.030^*^
Venous thrombosis	6 (1.0)	3 (1.5)	3 (1.1)	0 (0.0)	0 (0.0)	0.541
Sepsis	3 (0.5)	0 (0.0)	1 (0.4)	2 (1.8)	0 (0.0)	0.164
Hyperthermia	6 (1.0)	1 (0.5)	2 (0.8)	2 (1.8)	1 (2.4)	0.526
Urinary infection	21 (3.4)	5 (2.5)	8 (3.0)	5 (4.4)	3 (7.1)	0.420
Clavien-Dindo grade						
I	13 (2.1)	8 (3.9)	4 (1.5)	1 (0.9)	0 (0.0)	0.138
II	122 (19.6)	30 (14.7)	47 (17.8)	28 (24.6)	17 (40.5)	0.001^*^
III	15 (2.4)	6 (2.9)	6 (2.3)	3 (2.6)	0 (0.0)	0.722
IV	7 (1.1)	0 (0.0)	3 (1.1)	2 (1.8)	2 (4.8)	0.052
V	1 (0.2)	0 (0.0)	0 (0.0)	0 (0.0)	1 (2.4)	0.003^*^
Postoperative hospital stay, median (IQR), days	16.4 (14.0–20.0)	16.4 (14.0–20.0)	16.0 (14.0–20.0)	16.4 (14.0–20.0)	17.7 (15.0–21.8)	0.194

Values in parentheses are percentages

^*^Statistically significant (*P* < 0.05)

^a^Total complications were defined as any adverse event corresponding to Clavien–Dindo grade II or higher, occurring within 30 days after surgery. If a patient had more than one type of complication, the complication with the highest grade was recorded

^b^Severe complications were defined as Clavien–Dindo grade III or higher

WN, well-nourished non-visceral obesity; WO, well-nourished visceral obesity; MN, malnourished non-visceral obesity; MO, malnourished visceral obesity

Table 3 showed the univariate and multivariate logistic regression analyses of the factors associated with postoperative complications. The multivariate logistic analysis revealed that CCI (odds ratio [OR]: 2.343; 95% confidence interval [CI]: 1.573–3.491; P < 0.001), MN (OR: 1.952; 95% CI: 1.126–3.383; P = 0.017), and MO (OR: 4.112; 95% CI: 2.007–8.426; P < 0.001) were associated with postoperative complications.

**Table 3:** Univariate and multivariate logistic regression analyses for factors associated with postoperative complications

	Univariate analysis		Multivariate analysis
Tools	HR (95% CI) *P*		HR (95% CI) *P*
Age			
≥ 65/<65	1.488 (1.021–2.169) 0.039^*^		
Gender			
Male/ Female	0.925 (0.634–1.352) 0.668		
BMI			
< 18.5/18.5–23.9	2.317 (1.356–3.960) 0.002^*^		
≥ 24/18.5–23.9	0.940 (0.610–1.449) 0.780		
CCI			
≥ 1/0	2.219 (1.512–3.257) <0.001^*^		2.343 (1.573–3.491) <0.001^*^
ASA grade			
III/ I, II	1.781 (1.118–2.836) 0.015^*^		
Previous abdominal surgery			
Yes/No	1.569 (0.881–2.794) 0.126		
Tumor size			
> 4/≤4	1.018 (0.700–1.481) 0.925		
Tumor location			
Lower/ Upper	0.907 (0.575–1.431) 0.674		
Tumor differentiation			
Poorly/Well	1.368 (0.811–2.309) 0.240		
TNM stage			
II/ Tis, I	0.983 (0.591–1.635) 0.947		
III/ Tis, I	1.395 (0.882–2.205) 0.155		
Malnourished obesity			
WO/WN	1.256 (0.789–2.001) 0.336		
MN/WN	1.901 (1.106–3.268) 0.020^*^		1.952 (1.126–3.383) 0.017^*^
MO/WN	4.242 (2.097–8.581) <0.001^*^		4.112 (2.007–8.426) <0.001^*^

^*^Statistically significant (*P* < 0.05)

BMI, body mass index; CCI, Charlson Comorbidity Index; ASA, American Society of Anesthesiologists; TNM, tumor node metastasis; WN, well-nourished non-visceral obesity; WO, well-nourished visceral obesity; MN, malnourished non-visceral obesity; MO, malnourished visceral obesity

**Fig. 1:**
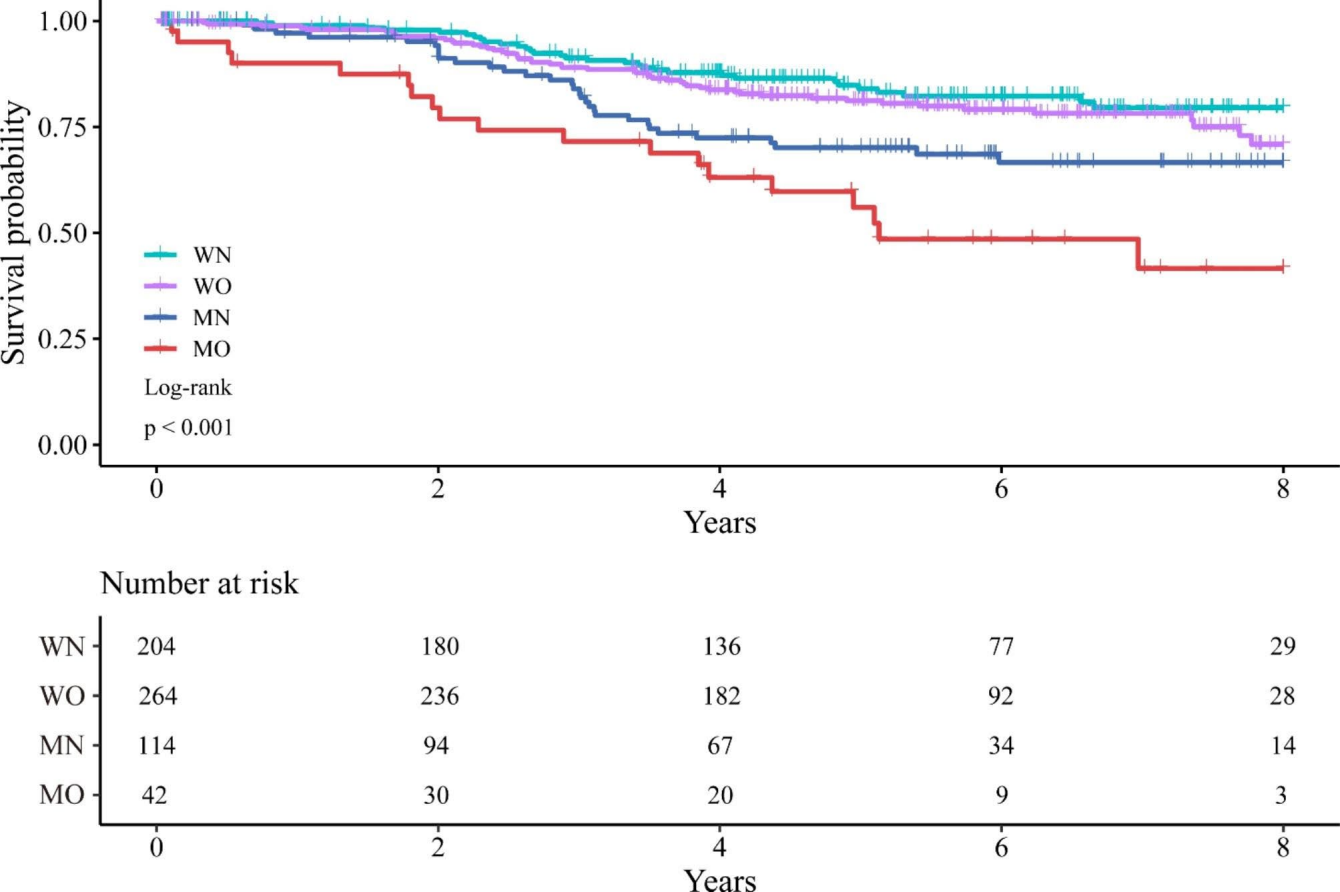
Kaplan-Meier curves for overall survival in rectal cancer patients according to the nutritional and visceral obesity status

The median follow-up time was 4.95 (3.38–6.72) years. A total of 133 deaths (21.3%) occurred during the follow-up, of which 117 were cancer-specific. Table 4 showed the univariate and multivariate cox regression analyses of the factors associated with OS. In the multivariate cox regression analysis, age (OR: 2.029; 95% CI: 1.358–3.034; P = 0.001), ASA grade (OR: 1.989; 95% CI: 1.350–2.929; P < 0.001), tumor differentiation (OR: 2.075; 95% CI: 1.336–3.223; P = 0.001), TNM stage ([II vs. Tis, I OR: 2.243; 95% CI: 1.222–4.118; P = 0.009] [ III vs. Tis, I, OR: 2.243; 95% CI: 2.310–7.003; P < 0.001]), and MO (OR: 2.615; 95% CI: 1.404–4.871; P = 0.002) were associated with worsened OS. Figure 1. showed the Kaplan-Meier curves for OS according to nutritional and visceral obesity status in patients with rectal cancer.

**Table 4:** Univariate and multivariate Cox regression analyses for factors associated with overall survival

	Univariate analysis		Multivariate analysis
Tools	HR (95% CI) *P*		HR (95% CI) *P*
Age			
≥ 65/<65	2.650 (1.818–3.863) <0.001^*^		2.029 (1.358–3.034) 0.001^*^
Gender			
Male/ Female	0.883 (0.626–1.246) 0.478		
BMI			
< 18.5/18.5–23.9	1.285 (0.796–2.075) 0.305		
≥ 24/18.5–23.9	0.635 (0.421–0.971) 0.036^*^		
CCI			
≥ 1/0	1.308 (0.917–1.864) 0.138		
ASA grade			
III/ I, II	2.767 (1.928–3.972) <0.001^*^		1.989 (1.350–2.929) <0.001^*^
Previous abdominal surgery			
Yes/No	1.148 (0.671–1.965) 0.614		
Tumor size			
> 4/≤4	1.241 (0.883–1.746) 0.213		
Tumor location			
Lower/ Upper	1.082 (0.727–1.610) 0.699		
Tumor differentiation			
Poorly/Well	2.324 (1.521–3.550) <0.001^*^		2.075 (1.336–3.223) 0.001^*^
TNM stage			
II/ Tis, I	2.469 (1.352–4.511) 0.003^*^		2.243 (1.222–4.118) 0.009^*^
III/ Tis, I	4.384 (2.528–7.603) <0.001^*^		4.022 (2.310–7.003) <0.001^*^
Chemotherapy			
Yes/No	1.036 (0.737–1.456) 0.837		
Malnourished obesity			
WO/WN	1.282 (0.822–2.000) 0.273		
MN/WN	1.961 (1.192–3.227) 0.008^*^		
MO/WN	3.759 (2.123–6.657) <0.001^*^		2.615 (1.404–4.871) 0.002^*^

^*^Statistically significant (*P* < 0.05)

BMI, body mass index; CCI, Charlson Comorbidity Index; ASA, American Society of Anesthesiologists; TNM, tumor node metastasis; WN, well-nourished non-visceral obesity; WO, well-nourished visceral obesity; MN, malnourished non-visceral obesity; MO, malnourished visceral obesity

**Fig. 2:**
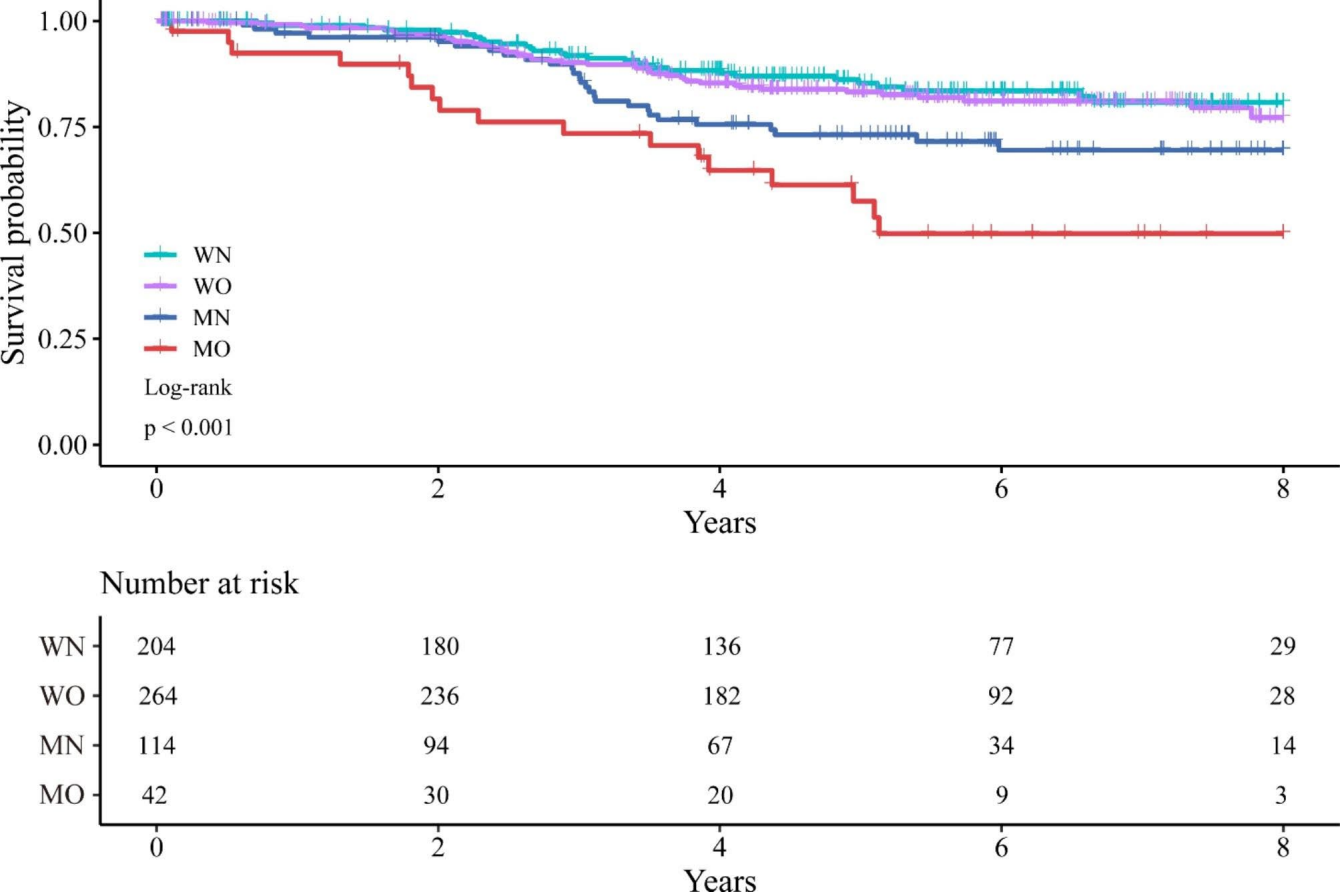
Kaplan-Meier curves for cancer-specific survival in rectal cancer patients according to the nutritional and visceral obesity status

Table 5 showed the univariate and multivariate cox regression analyses of the factors associated with CSS. In the multivariate cox regression analysis, age (OR: 1.913; 95% CI: 1.253–2.921; P = 0.003), ASA grade (OR: 1.792; 95% CI: 1.176–2.731; P = 0.007), tumor differentiation (OR: 1.938; 95% CI: 1.215–3.090; P = 0.005), TNM stage ([II vs. Tis, I OR: 9.920; 95% CI: 3.030–32.480; P < 0.001] [ III vs. Tis, I, OR: 20.158; 95% CI: 6.356–63.924; P < 0.001]), and MO (OR: 2.627; 95% CI: 1.363–5.063; P = 0.004) were associated with worsened CSS. Figure 2. showed the Kaplan-Meier curves for CSS according to nutritional and visceral obesity status in patients with rectal cancer.

**Table 5:** Univariate and multivariate Cox regression analyses for factors associated with cancer-specific survival

	Univariate analysis		Multivariate analysis
Tools	HR (95% CI) *P*		HR (95% CI) *P*
Age			
≥ 65/<65	2.461 (1.656–3.659) <0.001^*^		1.913 (1.253–2.921) 0.003^*^
Gender			
Male/ Female	0.923 (0.638–1.333) 0.668		
BMI			
< 18.5/18.5–23.9	1.125 (0.665–1.903) 0.661		
≥ 24/18.5–23.9	0.548 (0.346–0.868) 0.010^*^		
CCI			
≥ 1/0	1.063 (0.720–1.571) 0.758		
ASA grade			
III/ I, II	2.495 (1.685–3.696) <0.001^*^		1.792 (1.176–2.731) 0.007^*^
Previous abdominal surgery			
Yes/No	1.036 (0.570–1.882) 0.909		
Tumor size			
> 4/≤4	1.138 (0.790–1.639) 0.487		
Tumor location			
Lower/ Upper	1.082 (0.727–1.610) 0.699		
Tumor differentiation			
Poorly/Well	2.326 (1.483–3.646) <0.001^*^		1.938 (1.215–3.090) 0.005^*^
TNM stage			
II/ Tis, I	10.902 (3.338–35.604) <0.001^*^		9.920 (3.030–32.480) <0.001^*^
III/ Tis, I	21.858 (6.906–69.186) <0.001^*^		20.158 (6.356–63.924) <0.001^*^
Chemotherapy			
Yes/No	1.255 (0.873–1.805) 0.220		
Malnourished obesity			
WO/WN	1.155 (0.723–1.847) 0.546		
MN/WN	1.836 (1.087–3.101) 0.023^*^		
MO/WN	3.567 (1.959–6.493) <0.001^*^		2.627 (1.363–5.063) 0.004^*^

^*^Statistically significant (*P* < 0.05)

BMI, body mass index; CCI, Charlson Comorbidity Index; ASA, American Society of Anesthesiologists; TNM, tumor node metastasis; WN, well-nourished non-visceral obesity; WO, well-nourished visceral obesity; MN, malnourished non-visceral obesity; MO, malnourished visceral obesity

## Discussion

To the best of our knowledge, this is the first study to investigate the impact of visceral obesity and GLIM-defined malnutrition on short- and long-term outcomes in patients with rectal cancer. This study demonstrated that patients with both visceral obesity and GLIM-defined malnutrition were more likely to experience postoperative complications, poorer OS and CSS.

GLIM, a universal malnutrition model, was used in this study, in which the prevalence of GLIM-defined malnutrition was 25.0%. GLIM consisted of three phenotypical and two etiological criteria. In our study, cancer diagnosis was considered as the etiological criterion. GLIM-defined malnutrition was diagnosed when at least one of the phenotypic criteria was met. Non-volitional weight loss as a phenotypical criterion, was the most traditional indicator of malnutrition and was present in almost all malnutrition screening tools [21]. Skeletal muscle mass had attracted much attention in recent years. Many articles demonstrated the correlation between muscle mass and survival [19, 22]. When alternative phenotypical criteria were met, malnutrition might also be diagnosed despite the high BMI values when other phenotypical criteria were met. Thus, the GLIM criteria offer a major conceptual advancement in the diagnosis of malnutrition, even in patients with high BMI and adiposity. Different combinations of phenotypical and etiological criteria allowed for a wide range of GLIM applications [21]. Previous articles have pointed out that GLIM-defined malnutrition was a predictor of OS [23, 24]. Therefore, we used GLIM to evaluate the nutritional status.

While BMI was a convenient way to measure body weight, it failed to distinguish body composition, body fat distribution, fluid accumulation, and absolute weight gain among other factors [8, 25]. Therefore, visceral obesity was used in this study and the prevalence of visceral obesity was 50.8%. Many trials had demonstrated that visceral obesity was associated with longer operative time, greater blood loss during surgery, longer postoperative hospital stay, and higher postoperative complication rates after elective colorectal surgery [10, 11]. Furthermore, visceral obesity showed no influence on OS; however, it was significantly associated with disease-free survival in patients with resectable colorectal cancer [12]. However, it had not been verified in this article, which may be caused by our separation of the MO group from total visceral obesity group.

It was no surprise that TNM stage and tumor differentiation correlated with survival. In this study, CCI was a significant predictor of postoperative complication, but showed no influence on OS. Furthermore, ASA index was a significant predictor of OS; however, it showed no influence on postoperative complications. It showed that preoperative comorbidities were related to postoperative complications, while preoperative comorbidities that limited activity were related to OS. A low BMI score was associated with postoperative complications in the univariate analysis, but not in the multivariate analysis because it was one of the indicators for the diagnosis of malnutrition and was excluded as a confounding factor in the multivariate analysis. It had been reported that the association between BMI and colorectal cancer survival is U- or J-shaped [26]. In our study, high BMI was a protective factor in the univariate cox analysis and was excluded in the multivariate cox analysis. In this study, MN group was a protective factor for OS in the univariate cox analysis and was excluded in the multivariate cox analysis. It may be caused by the fact that we cut out the MO group from the malnutrition group.

With the growth of urbanization and industrialization in Asia, foods with low nutritional value were easily available and affordable, and sedentary lifestyles were promoted, contributing to the double burden of obesity and malnutrition [13]. Our study showed that visceral obesity and malnutrition were well evaluated using CT and GLIM. How to intervene in it was also a challenge. Previous studies have indicated that preventing malnutrition should be a part of the obesity medical care plan, which included a lifestyle change, making the right food choices and avoiding unhealthy foods [27]. Moreover, fortified foods such as specific vitamin supplements should be included to prevent shortages. It was not widely accepted that multi-model pre-habilitation should include exercise therapy, nutritional supplementation, and hematologic optimization preoperatively [28]. Further research was needed to explain the factors shaping MO in patients, as well as appropriate treatment strategies.

This study had some limitations that should be considered. Firstly, despite the attempts to minimize the confounding factors, the retrospective nature of our analysis posed a risk of selection bias. Secondly, we are supposed to keep in mind whether our cut-points are appropriate to define low SMI and visceral obesity. Several studies have established definitions for low SMI and visceral obesity according to different criteria [11, 29, 30]. Owing to the lack of a uniform age-specific threshold for CT measured low SMI and visceral obesity, we applied a unified cutoff value for SMI based on our previous study [19] and cutoff values for visceral obesity based on the Japanese research [20]. There still remained the need for definitive criteria of CT measured low SMI and visceral obesity for Asian. Finally, this was a single-center study among Chinese patients with rectal cancer, which may not be applicable to other ethnic populations and regions. In the future, a multicenter prospective study in different populations is required to validate our findings.

## Conclusion

In conclusion, this study demonstrated that the combination of visceral obesity and malnutrition resulted in higher postoperative complication and mortality rates and was a good indicator of poor prognosis in patients with rectal cancer. According to our findings, visceral obesity and malnutrition had a double burden. Therefore, health lifestyle programs and policies are required to reduce visceral obesity and promote nutritional health.

## Data Availability

The datasets generated during and analyzed during the current study are not publicly available, because they contain information that could compromise the privacy of research participants, but they are available from the corresponding author on reasonable request.
